# Electrodeposition of Two-Dimensional Pt Nanostructures on Highly Oriented Pyrolytic Graphite (HOPG): The Effect of Evolved Hydrogen and Chloride Ions

**DOI:** 10.3390/nano8090668

**Published:** 2018-08-28

**Authors:** Mario A. Alpuche-Aviles, Filippo Farina, Giorgio Ercolano, Pradeep Subedi, Sara Cavaliere, Deborah J. Jones, Jacques Rozière

**Affiliations:** 1 Department of Chemistry, University of Nevada, Reno, NV 89557, USA; psubedi@unr.edu; 2 Institute Charles Gerhardt Montpellier, Laboratory of Aggregates Interfaces and Materials for Energy, University of Montpellier, 34095 Montpellier, France; filippo.farina@umontpellier.fr (F.F.); giorgio.ercolano@umontpellier.fr (G.E.); deborah.jones@umontpellier.fr (D.J.J.); jacques.roziere@umontpellier.fr (J.R.)

**Keywords:** electrodeposition, platinum, highly oriented pyrolytic graphite, 2D growth

## Abstract

We discuss the electrodeposition of two-dimensional (2D) Pt-nanostructures on Highly Oriented Pyrolytic Graphite (HOPG) achieved under constant applied potential versus a Pt counter electrode (E_appl_ = ca. −2.2 V vs. NHE, normal hydrogen electrode). The deposition conditions are discussed in terms of the electrochemical behavior of the electrodeposition precursor (H_2_PtCl_6_). We performed cyclic voltammetry (CV) of the electrochemical Pt deposit on HOPG and on Pt substrates to study the relevant phenomena that affect the morphology of Pt deposition. Under conditions where the Pt deposition occurs and H_2_ evolution is occurring at the diffusion-limited rate (−0.3 V vs. NHE), Pt forms larger structures on the surface of HOPG, and the electrodeposition of Pt is not limited by diffusion. This indicates the need for large overpotentials to direct the 2D growth of Pt. Investigation of the possible effect of Cl^−^ showed that Cl^−^ deposits on the surface of Pt at low overpotentials, but strips from the surface at potentials more positive than the electrodeposition potential. The CV of Pt on HOPG is a strong function of the nature of the surface. We propose that during immersion of HOPG in the electrodeposition solution (3 mM H_2_PtCl_6_, 0.5 M NaCl, pH 2.3) Pt islands are formed spontaneously, and these islands drive the growth of the 2D nanostructures. The reducing agents for the spontaneous deposition of Pt from solution are proposed as step edges that get oxidized in the solution. We discuss the possible oxidation reactions for the edge sites.

## 1. Introduction

The use of Pt is of interest in many renewable energy applications, namely, in the use of technologies that convert chemical energy to electricity, such as proton exchange membrane fuel cells (PEMFCs). Because of the low abundance and high cost of this noble metal, research has centered on the use of Pt nanostructures on a highly conducting and porous support, conventionally carbon black, with the goal of using a minimum loading while preserving high electroactivity towards the oxygen reduction reaction (ORR, the sluggish reaction taking place at the cathode side) and long-term stability. The strategies to minimize the Pt amount call for a shift from monometallic nanoparticles (NPs) to the use of nano-engineered architectures with tailored morphologies and compositions [1,2]. For instance, bi- or tri-metallic particles, alloys [3,4,5,6] and successively de-alloyed [7] structures where Pt is associated with other transition metals (e.g., Ni, Co, Cu) have been prepared and demonstrated high ORR activity and durability. Another very promising class of tailored electrocatalysts is represented by core@shell nanostructures with a thin Pt skin covering a transition metal core in 0D (particle-like) [8,9,10] and 1D (fiber-like) morphologies [11,12,13,14,15,16,17]. The advantage of a Pt thin layer morphology on other non-metallic supports (carbon, polymers) has been extensively demonstrated to maximize Pt exploitation by minimizing the contribution of edge/corner sites and to enhance its stability by circumventing any possibility of nanoparticle aggregation [18,19,20]. In particular, Pt extended surfaces deposited on high aspect-ratio materials such as nanofibers [19,21] and whiskers [22] presented exceptional increase of the ORR specific activity, which was kept after prolonged electrochemical cycling. Among the methods being investigated to produce Pt conformal thin films, atomic layer deposition (ALD), electrochemical atomic layer deposition (EC-ALD), pulsed laser deposition, surface-limited redox replacement (SLRR), galvanic displacement [18,21,23,24] and other vacuum techniques, such as magnetron sputtering are employed [25]. 

The goal of electrodepositing Pt extended surfaces in a continuous and conformal fashion on the support is complicated by the fact that the 3D growth of this metal is favored with the formation of dendrites [26] and flower-like agglomerates. It was previously reported that it is possible to prepare Pt thin films on flat metallic Au surfaces via a self-limiting electrodeposition process performed at high overpotential [27]. More recently, some of us reported the electrodeposition of thin Pt nanostructures by pulsing the electrodeposition at high overpotentials on carbon fiber webs obtained by electrospinning of precursor solutions [19,28]. The material obtained was determined to be 2D contiguous Pt nanoplatelets. The resulting self-supported nanofibrous electrode (NFE) was demonstrated to be an efficient and stable ORR material. The understanding of the mechanistic aspects that yield the electrodeposition of a Pt thin film on a carbonaceous model surface, such as Highly Oriented Pyrolytic Graphite (HOPG), is crucial for the deposition of ultra-low and continuous coverage of Pt on carbon supports of different morphology and porosity [29].

In this paper, we focus on the study of the electrodeposition step in the first cycle of the electrodeposition sequence (200 s). We address the mechanistic aspects that direct the growth of a 2D film of Pt on HOPG as a model for the deposition of Pt on carbon. The effect of electrolyte concentration and potential on Pt morphology has been recognized for some time [30], and studies include the use of HOPG in mechanistic studies [30,31,32]. However, up to now, the electrodeposition of Pt has been studied at much lower overpotentials than those used in this work. Penner and coworkers proposed the electrodeposition of Pt nanoparticles with homogeneous size distribution [30]. Simonov et al. [33] revised the conventional model of nucleation and growth [26] and distinguished (i) a primary nucleation of Pt on the substrate, (ii) a secondary nucleation around the Pt deposits, and (iii) the growth phase around the Pt deposits. Here we present evidence that spontaneous deposition of Pt when the HOPG substrate immersed at open circuit, that is, without an external current flowing through the substrate, is a critical initial step. The initial stages of Pt deposition continue to be the subject of investigation because they are thought to control the deposition process. For Pt on HOPG, the spontaneous deposition of Pt has been observed before [30,32,34,35,36] and although the process is not well understood, it can nonetheless control the size and distribution of the Pt nanostructures deposited during a subsequent potentiostatic step. Zoval et al. first reported this electroless deposition of Pt [30], which led to a decrease in the reproducibility of electrodepositing Pt NPs on HOPG. They used an anodic potential step (ca. 0.8 V vs. NHE for 1 min) to prevent the spontaneous deposition of Pt at open circuit potential (OCP) before the potentiostatic NP deposition. They reported that the oxidizing treatment prevented the spontaneous deposition of Pt at OCP and made the Pt NPs deposited during the subsequent potentiostatic step more homogenous.

Similarly, Lu and Zangari [31] applied an anodic treatment to the substrate to isolate the nucleation and growth during electrodeposition from the electroless deposition of Pt. Later, they addressed the spontaneous deposition of Pt from a 1 mM H_2_PtCl_6_ solution without additional excess electrolyte and in 0.1–0.2 M HCl. They found that the Pt deposits preferentially on edges although it can also deposit on terraces, regardless of the use of electrolyte. The Pt islands from the spontaneous process lead to dendritic growth of Pt, and thus, they used an oxidation pretreatment (1 V vs. NHE for 2 min) for the HOPG to avoid the spontaneous deposition and to obtain monodispersed Pt NPs [32]. Arroyo-Gómez and García [34] showed that the NPs cover the HOPG surface completely in a solution of 1 mM H_2_PtCl_6_ + 0.05 M H_2_SO_4_. Under these conditions, without excess Cl^-^, the precursor hydrolyzes quickly [31,33] at RT. Initially, the Pt deposits on edges and at longer times the deposition occurs on the terraces along with larger Pt particles on the surface. After 2 h of immersion the substrate electrocatalytic activity approached that of bulk Pt. The electroless process occurs with [30] or without excess electrolyte [32] (Cl^−^ [30] or SO_4_^2−^ [34]) and in a nonaqueous electrolyte containing chloride (2.5 mM H_2_PtCl_6_ + 80 mM Et_4_NCl in CH_3_CN) [35]. In the nonaqueous electrolyte, oxidizing the HOPG surface before immersion in the Pt solution yielded a more uniform NP electrodeposit.

Since the original report, the observation that oxidizing the substrate minimizes the spontaneous deposition of Pt/HOPG has led to the conclusion that a partially oxidized site on the surface is the reducing agent. This reducing agent has been ascribed to defects [30], oxidized [30] and hydrogenated [37] sites on the carbon surface. However, there are no reports on the half oxidation reaction that reduces the Pt precursor. Interestingly, the electrochemical papers lack X-ray photoelectron spectroscopy (XPS) studies of the surface oxidation and, to get insight into the half reactions, we look at the surface energies and composition studies of the spontaneous deposition of Au on HOPG from solutions [38,39,40]. Here, we propose that edge sites oxidize sequentially to C–OH and C=O groups, based on analogous studies for the deposition of Au on HOPG to produce gas-phase catalysts and our own results that see preferential deposits on the edges, in agreement with previous reports [32,34,35]. We address the effect of the initial electroless deposition of Pt on the formation of 2D nanostructures on HOPG. We point out that in the previous reports, the focus was to electrodeposit NPs, but our goal is the production of a conformal film. Despite evidence that the spontaneous deposition of Pt occurs, we do not see dendritic growth, which indicates that another process limits the growth of the initial deposits. Therefore, we discuss both stages of the Pt deposition, the potentiostatic mode that grows the 2D deposit and the initial electroless deposition.

## 2. Materials and Methods 

Reagents and Materials: HOPG (ZYH grade, 12 × 12 mm from Veeco-Bruker, Camarillo, CA, USA) was used as the working electrode in a two or three electrode cell. The working electrode was freshly exfoliated before every electrochemical experiment and immediately masked with Kapton^®^ tape (RS Components SAS, Beauvais, France). The electrode area exposed to the solution was typically 6 × 6 mm. The backside of the HOPG was taped to a Pt wire or Cu tape, keeping the solution from being exposed to the Pt or Cu. All the reagents used are of analytical grades and used without purification: H_2_PtCl_6_ (ACS reagent, 37.50% Pt basis) and NaCl were purchased from Sigma-Aldrich (St. Louis, MO, USA). All aqueous solutions were prepared in 18 MΩ⋅cm water. 

Characterization: The samples were characterized with tapping mode atomic force microscopy (TM-AFM). A Bruker Nanoman AFM instrument (Bruker SAS, Palaiseau, France) with a Nanoscope 5 controller was equipped with the following tips: a silicon Point Probe Plus (Nanosensors, Neuchâtel, Switzerland) with 6.5 N/m force constant and f = 157 kHz resonance. The images were processed with Gwyddion version 2.44 (Czech Metrology Institute, Brno, Czech Republic) both for image flattening and thickness measurements. 

Electrochemical Measurements: Oxidation currents were positive following the IUPAC convention. Experiments were performed with either a Bio-Logic^®^ SP 300 (Bio-Logic SAS, Seyssinet-Pariset, France) potentiostat (2 channels configuration) or a CH Instruments CHI 760D and 700D workstation (CH Instruments Inc., Austin, Texas, USA). The working electrode was usually an HOPG setup, although some controlled experiments were performed with a 1.6 mm Pt disk electrode. The counter electrode was either a graphite sheet (5 × 5 cm) glassy carbon sheet or a graphite rod (Alfa Aesar, Heysam, UK). We did not use Pt CE in experiments performed in Pt-free solutions. The reference electrode was an Ag/AgCl reference electrode (Fisher Scientific, Illkirch, France; E = 0.197 V vs. NHE) used with a bridge filled with 0.5 M NaCl with the pH adjusted to 2.3, as in the electrodeposition solution. The standard rate constants, k^0^ were determined by fitting experimental data to an electrochemical mechanism [41]. The linear sweep voltammograms (LSVs) obtained for the H^+^ reduction where simulated with the commercial software DigiElch version 7.FD (ElchSoft, Kleinromstedt, Germany) that allowed us to correct the LSVs for iR_u_ drop and capacitive charging. The k^0^ value was varied until a good fit of the experimental data was obtained using previously reported values of diffusion coefficients of H^+^ and H_2_ as well as the proton concentration. (D_H_^+^ = 7.1 × 10^−5^ cm^2^/s [42] and for H_2_, D_H_2__ = 3.37 × 10^−5^ cm^2^/s) [43]. 

Pt/HOPG Electrode preparation: Full details are given elsewhere for the preparation of the Pt electrode on HOPG and [19,29]. Briefly, the HOPG was exposed to the solution, and the deposition was performed either on a 2- or 3-electrode cell. In the 2-electrode cell, Pt electrodeposition is performed by applying −3 V vs. the counter electrode for 200 s. This potential was applied immediately following an immersion time during which the solution was cooled to 4 °C while bubbled with nitrogen to remove oxygen. The solution was stirred at 900 rpm during electrodeposition to remove hydrogen bubbles evolving from the surface. The electrodeposition of Pt was performed from the following solution: 3 mM H_2_PtCl_6_, 0.5 M NaCl with the pH adjusted to 2.3 with HCl. 

## 3. Results and Discussion

### 3.1. Electrodeposition Process Characterization 

**Figure 1:**
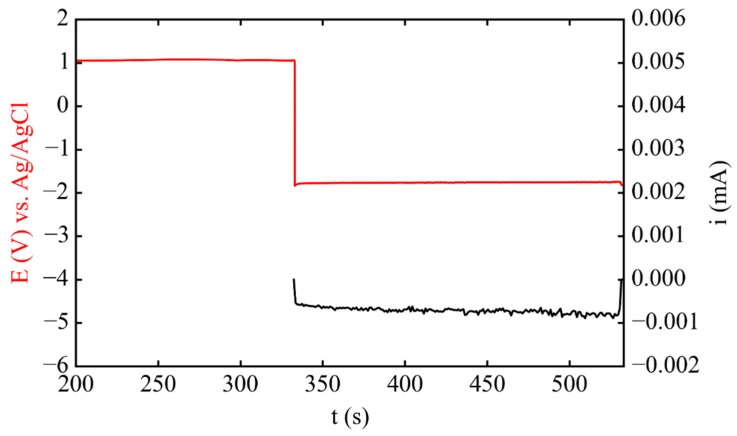
Electrodeposition Pt on Highly Oriented Pyrolytic Graphite (HOPG) (0.36 cm^2^) in 3 mM H_2_PtCl_6_, 0.5 M NaCl, pH 2.3, under N_2_ bubbling and 600 rpm stirring. The time between 200 and 320 s corresponds to the sample immersed at OCP = 1 V vs. Ag/AgCl = 0.8 V vs. NHE. At 320 s, E_app_ = −3 V vs. CE, which is ca. −2 V vs. Ag/AgCl and −2.2 V vs. NHE.

Figure 1 shows the data of the electrodeposition on HOPG. The electrodeposition was performed in a two-electrode cell, with the first channel of the Bio-Logic potentiostat (floating configuration) with the potential simultaneously monitored with the second potentiostat channel. Initially, the electrode is immersed in the solution and kept under OCP conditions, which is typically around +1 V vs. Ag/AgCl (ca. 0.8 V vs. the normal hydrogen electrode, NHE). After the immersion time, a constant potential of −3 V vs. the CE is applied, which corresponds to ca. −2 V vs. Ag/AgCl (−2.2 V vs. NHE). At these potentials, the current is around 1 μA, which corresponds to ca. 2.8 μA/cm^2^. We focus our discussion on two aspects: (1) the initial OCP value and (2) the value of the applied potential (E_app_) to evaluate the effect of eventual hydrogen formation.

### 3.2. Phase 1: Electrode Immersion 

The potential achieved for the HOPG immersed in the Pt solution (Figure 1) is similar to that required for the Pt electrodeposition. Because the solution contains PtCl_6_^2−^, the electrodeposition should be a sequential process with the ultimate production of Pt^0^ according to the following reactions [44]:

PtCl_6_^4‒^_(aq)_ + 2e → PtCl_4_^2‒^_(aq)_ + 2Cl^‒^_(aq)_     E^0^ = 0.726 V (1)

**Figure 2:**
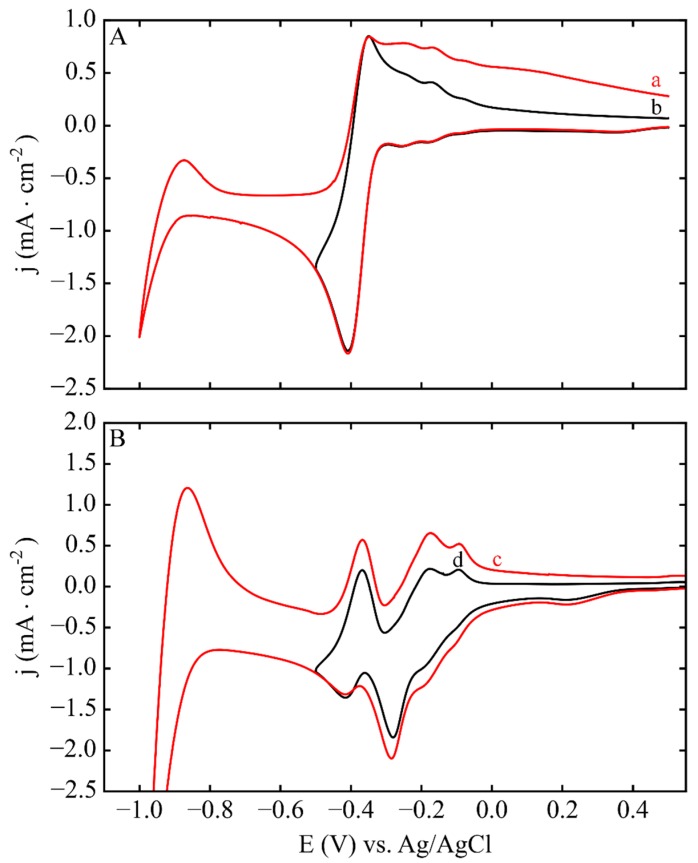
Cyclic voltammetry of a 1.6 mm Pt electrode in (**A**) 0.5 M NaCl pH 2.3 (no Pt) and (**B**) in the same electrolyte in (A) +3 mM H_2_PtC_l6_. CVs (a,c) and (b,d) were recorded in different potential windows, ν = 50 mV/s.

PtCl_4_^2‒^_(aq)_ + 2e → Pt + 4Cl^‒^_(aq)_        E^0^ = 0.758 V (2)

where all potentials are with respect to NHE. However, the potentials reported in Equations (1) and (2) are derived from complexation schemes; they are normally not observed under usual experimental conditions [45]. The potentials observed in Figure 1 during the incubation period at OCP are similar to those used for Pt deposition, and in fact, in different experiments, we observed potentials more negative than those in Equations (1) and (2). The deposition conditions were investigated by cyclic voltammetry. In Figure 2 are presented the CVs of a 1.6 mm Pt disk electrode immersed blank (without Pt, A) and in the deposition solution (B). The black and red traces correspond to experiments performed with different potential scans at the same scan rate (ν). Concerning the experiments on HOPG in the absence of Pt, the peak around −0.4 V vs. Ag/AgCl (Figure 2A) is attributed to the diffusion limited H^+^ reduction to H_2_. In the region between −0.4 V and 0.3 V vs. Ag/AgCl, several features in the CV appear when Pt is introduced in the deposition solution. Note that these potentials correspond to −0.2 to 0.5 V vs. NHE, which are positive to the potentials in Equations (1) and (2). Note also that although the step-wise reduction of Pt^4+^ to Pt^0^ is expected, the CV shows that the mechanism is more complicated than two consecutive 2-electron processes. Uosaki et al. reported the sequential 2-electron reduction in H_2_SO_4_ electrolyte with peak potentials observed on Pt(111) in agreement with the values of Equations (1) and (2) [46]. However, in Cl^−^ electrolytes, the reduction potentials shift to negative values without resolving the two reduction steps on the CV [31]. We will not attempt to discuss here the mechanism of Pt^4+^ reduction, but will use the CVs of the electrode materials in different electrolytes to explain the formation of 2D Pt structures. At negative potential regions (E < 0.7 V vs. NHE), on Pt, the deposition of Pt from one of the PtCl*_x_* complexes is expected.

**Figure 3:**
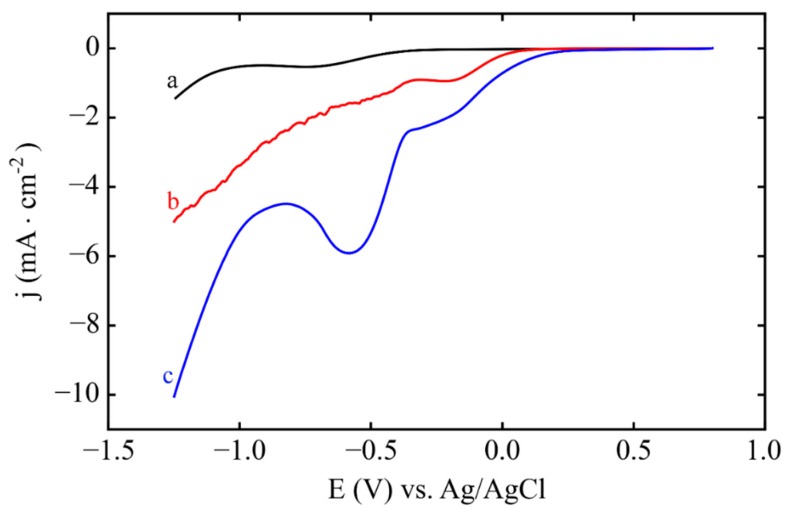
Linear sweep voltammetry of 3 independently prepared HOPG surfaces in 3 mM H_2_PtCl_6_ 0.5 M NaCl pH 2.3 (a–c), ν = 100 mV/s.

The CV for HOPG in the Pt deposition solution is a strong function of the nature of the HOPG surface. Figure 3 depicts the linear sweep voltammetry (LSV) of three independently prepared HOPG surfaces in the same electrodeposition solution as the samples shown in Figure 2B. Interestingly, the electrodeposition on HOPG does not display a peak for the diffusion-limited deposition of Pt/HOPG and only the peak for H_2_ evolution is present at E = −0.5 V, but no peak for Pt deposition is seen. At potentials more negative than 0.3 V vs. Ag/AgCl, or 0.5 V vs. NHE Pt deposition on HOPG is again apparent. However, the rates of deposition vary widely, and we propose that this is due to the different conditions of the surface exposed by cleavage of the C–C stacking with tape.

**Figure 4:**
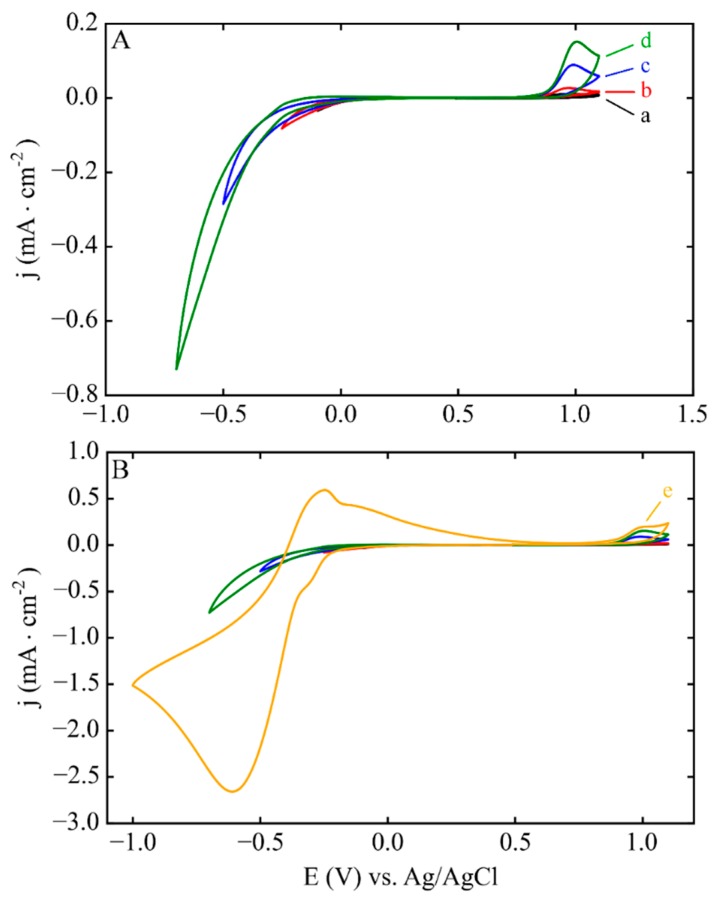
(**A**) Sequence of cyclic voltammograms (CVs) of Pt deposition on the same HOPG surface (a–d), ν = 50 mV/s. The CVs increase the potential window in the negative direction. (**B**) Shows the same CVs in (**A**) for comparison with the final CV (e).

We further investigated the electrodeposition of Pt on an HOPG surface by cyclic voltammetry. Figure 4 depicts a series of sequential CVs obtained on the same surface. Note that the scan rate here is twice that in Figure 3, to minimize the time in which the surface is modified during the polarization. Also, each sequential CV was recorded on increasing the potential window (a–e), but no peak characteristic of the deposition of Pt from the 3 mM solution of Pt precursor was observed. After 4 consecutive CVs, the peak for H_2_ evolution in the solution is seen (curve e). An anodic peak appears around +1.0 V vs. Ag/AgCl, or 0.8 V vs. NHE, which is consistent with the oxidation of Pt to a PtCl*_x_* complex. The peak intensity increases with the number of CVs, in agreement with a higher amount of Pt present on the surface. This result suggests that at potentials more negative than 0.8 V vs. NHE, the Pt deposit is stable on the HOPG surface. However, the CVs or LSVs do not reach a diffusion-limited rate for the deposition of Pt/HOPG.

**Figure 5:**
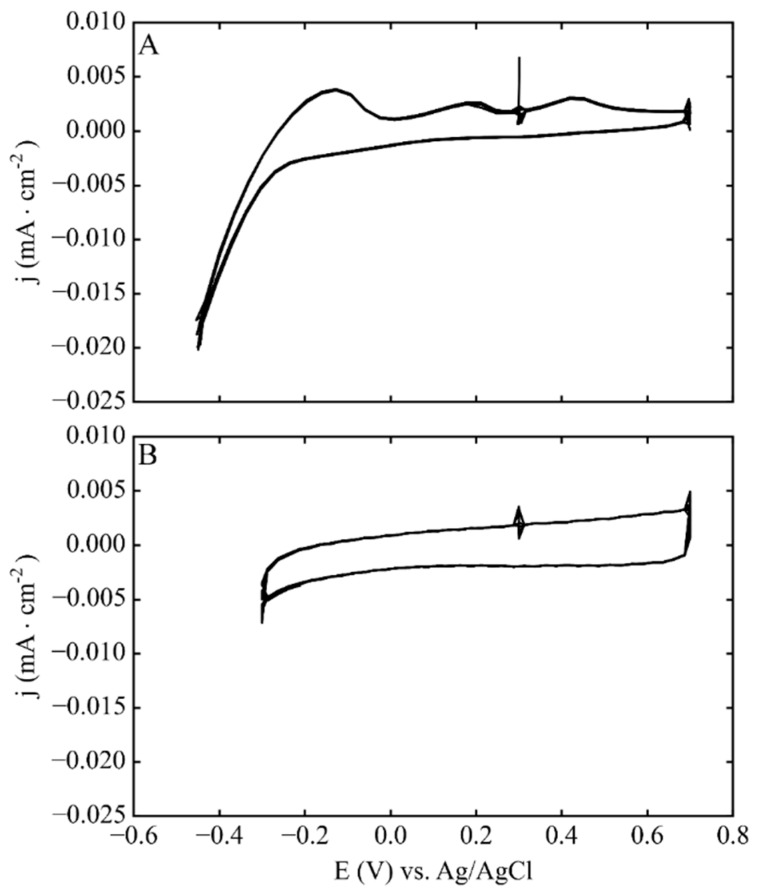
(**A**) CVs of a HOPG electrode in 0.5 M H_2_SO_4_ exposed to the electrodeposition solution (3 mM H_2_PtCl_6_, 0.5 M NaCl, pH 2.3) for 200 s. The electrode was immersed but not connected to the external circuit during the exposure to the Pt precursor, then rinsed, dried and characterized in 0.5 M H_2_SO_4_. (**B**) Behavior of a freshly cleaved HOPG electrode without exposure to the Pt deposition solution.

The results in Figure 3 and Figure 4 indicate that the Pt deposit is stable on HOPG at potentials that are consistent with the bulk reduction potentials for the complexes in Equations (1) and (2). However, the OCP before deposition indicates that Pt can be formed when the HOPG substrate is immersed in the precursor solution. To investigate this possibility, we immersed a freshly-cleaved HOPG electrode in the electrodeposition solution without an applied potential, and without a connection to an external circuit, and then rinsed it with water. The voltammetry of this electrode in 0.5 M H_2_SO_4,_ and, for comparison, the behavior of HOPG in the same solution, are presented in Figure 5A,B, respectively. The CV of the HOPG exposed to the Pt precursor solution shows the characteristic features for H_2_ evolution, which is consistent with the formation of Pt on the surface. Note that the activity is relatively low, since the peaks for hydrogen adsorption and desorption are not well defined. Recently, Arroyo-Gomez and Garcia reported higher electrocatalytic activity for spontaneously deposited Pt on C with electroactivity being a function of time [34]; the highest activity was observed after 2 h of exposure in a solution at room temperature. In the present study, the incubation was performed at low temperatures (4–5 °C), that we propose slow down the deposition of Pt, which has been reported to be a slow process even at room temperature [34]. 

**Figure 6:**
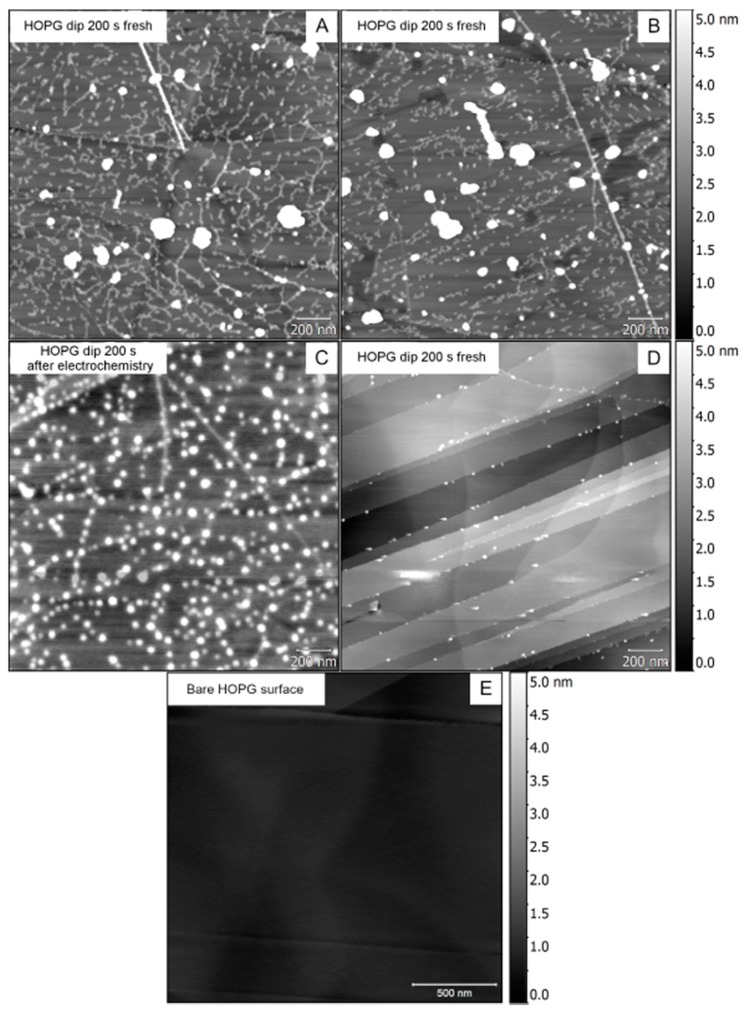
Atomic force microscopy (AFM) images of HOPG surfaces immersed in a Pt deposition solution (3 mM H_2_PtCl_6_, 0.5 M NaCl, pH 2.3) recorded after the immersion (**A**, **B** and **D** fresh) and after the electrochemical cycles reported in Figure 5 (**C**, after electrochemistry). All the electrodes were prepared by immersion at open circuit potential (OCP), i.e., without applying an external potential. (**E**) Corresponds to a blank HOPG.

The AFM images obtained on an identically prepared electrode, exposed to the Pt deposition solution but without a bias for electrodeposition (OCP immersion), are shown in Figure 6. Pt structures have formed on the surface of the HOPG and, interestingly, while they appear to be dispersed on the surface, the features follow the defects on HOPG. Spontaneous deposition on HOPG is not well understood and, while edges and defects on HOPG have been suggested to act as reducing agents [30], there are reports of no preferential deposition around the edges [32]. Our observations support that the defects on HOPG are responsible for the deposition of Pt, (Figure 6D which shows the detail of step edges) which occurs spontaneously once the HOPG is immersed in a Pt-containing solution. Compare with Figure 6E which is the AFM of bare HOPG. The Pt deposition is occurring preferentially on the step/edges of the HOPG substrate and other defects around the surface. One sample was subjected to electrochemical cycling represented in Figure 5, and the AFM of this sample is shown in Figure 6C after electrochemical testing. Figure 6C shows that the Pt structures on the HOPG surface become larger NPs, probably due to the reconstruction of the initial Pt nanostructures during the CVs. This observation suggests that the Pt structures spontaneously deposited on the surface while electrochemically active (Figure 5) are not strongly attached to the HOPG surface because they re-construct (compare Figure 6A,B,D before and after electrochemistry, Figure 6C) and also we observed that the AFM would move some particles during imaging.

**Scheme 1:**
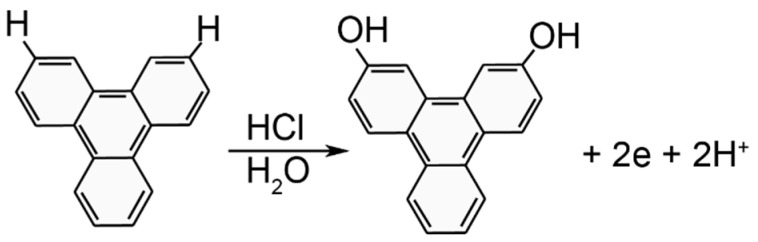
Proposed oxidation of the HOPG step edges in deposition solution 3 mM H_2_PtCl_6_ 0.5 M NaCl pH 2.3. Triphenylene illustrates a terminal section of the extended HOPG structure. The reaction is deduced from the data in ref [39].

The spontaneous Pt deposition that we observed here also occurs under a wide range of conditions [30,32,34,35,36]. As discussed in the introduction, partially oxidized sites have been proposed as the reducing agent for Pt. Penner and co-workers proposed the oxidation of step edge and other defect sites that could be aldehydes, alcohols and ketons partially oxidized [30]. This assignment was made based on the observation that surface enhanced Raman spectroscopy detect carbonyl functionalities on thermally pitted HOPG substrates. We are not aware of reports on the half oxidation reaction during Pt deposition in the literature, in part because the spontaneous deposition of Pt on C is a point that has received limited attention in the literature. Because the electrochemical papers lack XPS studies of the surface oxidation before and after Pt deposition, we get insight into the half reactions of oxidation from the XPS studies of the adsorption and deposition of Au on HOPG from acidic solutions [38,39,40]. Because they do not report the redox properties of these surface reactions, we must deduce possible reactions from surface composition studies. XPS showed that immersing HOPG in aqueous HCl (diluted) yields mostly an OH-terminated surface [39] together with a theoretical study to support the experimental finding. Density functional theory (DFT) calculations of the step edges along the HOPG terminated on a C–OH group gave the more stable configuration having the OH group roughly in plane with the graphitic sheet oriented towards the H on the same C=C bond of the armchair edge [39]. Because this configuration has a slightly higher energy than a single graphene sheet (by 0.035 eV/A) the authors proposed that mild oxidation conditions are necessary to produce the C–OH groups on the edges. During heating, the hydroxyl groups oxidize to produce ketone and ester groups on the HOPG surface [39,40]. However, the C–OH groups reduced Au^3+^ to Au^0^ from solution while the C–OH oxidize to C=O groups [38]. Therefore, based on the findings by XPS and AFM of Au deposition on HOPG [38,39,40] along with the AFM studies in this work (Figure 6A,B,D) and previous reports that show preferential deposition of Pt along the step edges [32,34,35], we propose the following oxidation reactions in our precursor solution. Initially, during immersion of HOPG to the precursor solution (3 mM H_2_PtCl_6_ 0.5 M NaCl pH 2.3, which corresponds to dilute HCl), the step edges oxidize to C–OH groups as shown in Scheme 1. To illustrate the reaction, we use a triphenylene to depict the step edges on the HOPG surface that yields an OH and H on the same C=C bond as in the DFT study by Buono et al. [39]. The structure also affords the simplest oxidation case, where the half-reaction is a 2e, 2H^+^ oxidation without the need to create radicals or dangling bonds on the polycyclic structure during the oxidation of the C–H edge sites to C–OH or for the following oxidation of the OH groups.

**Scheme 2:**
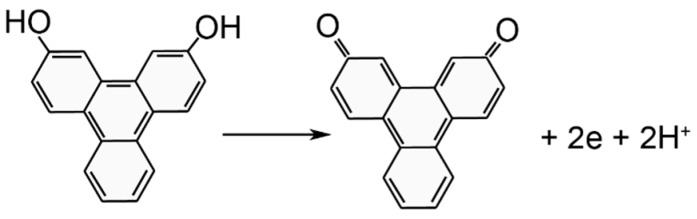
Proposed oxidation of HOPG step edges as reducing agents for H_2_PtCl_6_ in the precursor solution. Derived after ref. [38], our results and other reports [32,34,35] that find preferential Pt deposits along the edges.

We propose that a second step is the oxidation of the C–OH to C=O. The more likely redox reaction here is the 2e, 2H^+^ oxidation depicted in Scheme 2, with adjacent C–OH groups that can provide enough electrons to reduce the precursor to Pt^0^ either by the two sequential, 2e reactions in Scheme 1 and Scheme 2 or through the oxidation of adjacent groups.

### 3.3. Phase 2. Electrodeposition 

**Figure 7:**
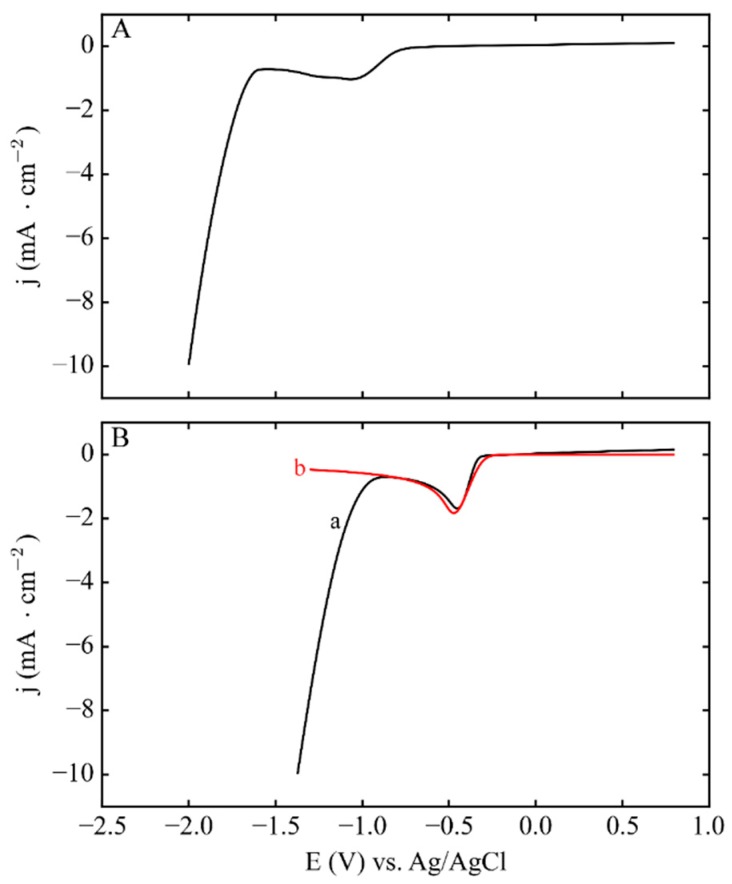
(**A**) Linear sweep voltammograms (LSV) of a bare HOPG electrode in 3 mM H_2_PtCl_6_, 0.5 M NaCl, pH 2.3, under N_2_ and (**B**) For a Pt/HOPG electrode, same conditions as in (**A**). Line (a) shows the experimental data and line (b) the comparison to the digital simulations.

**Figure 8:**
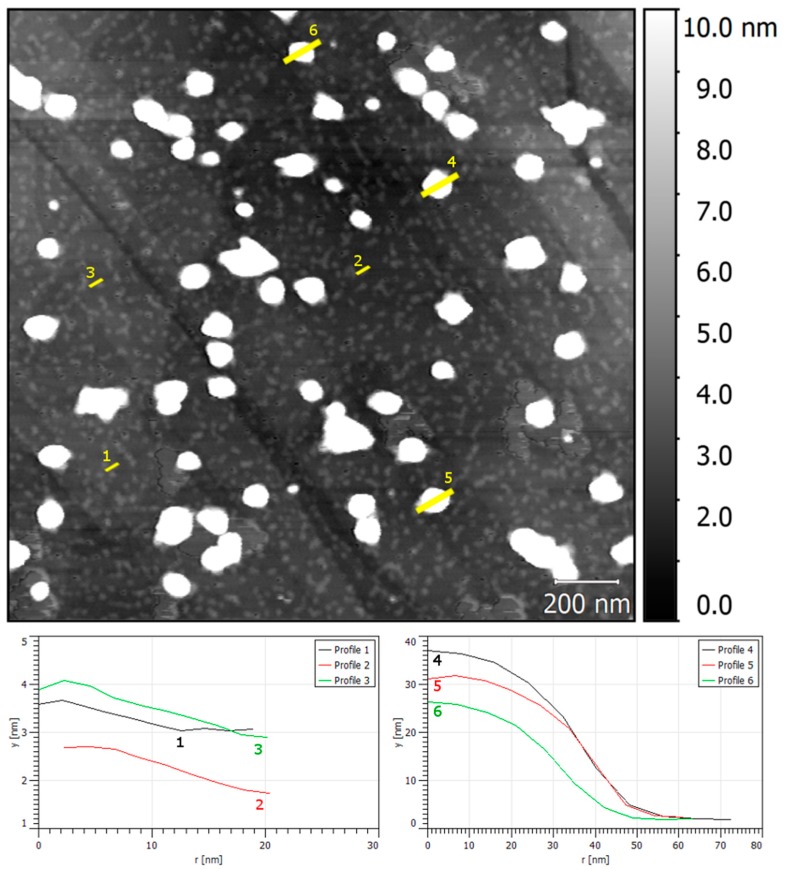
AFM of a sample electrodeposited at −0.5 V vs. Ag/AgCl in 3 mM H_2_PtCl_6_, 0.5 M NaCl, pH 2.3. Radial profiles of the two population of the Pt features are also reported.

The results seen on Figure 3 and Figure 4 on the HOPG, compared with those obtained with the Pt electrode (Figure 2), indicate that reduction of PtCl_x_ complexes is an intrinsically slower process on the surface of HOPG than on Pt. Therefore, the deposition of Pt is expected to occur at a higher rate on Pt structures than on a bare HOPG surface, leading to tridimensional structures. Eventually, at high enough overpotentials, the production of bulk H_2_ becomes the predominant electrolytic process in the deposition system. Therefore, we investigated the effect of H_2_ on the deposition of the sample. In Figure 7 are shown the LSVs obtained for a freshly cleaved HOPG sample and Pt/HOPG sample, prepared under the conditions of Figure 1. Figure 7A is the data for bare HOPG in the blank electrolyte, note that there is a small peak around −1 V vs. Ag/AgCl, which indicates negligible H^+^ reduction activity for HOPG. Figure 7B shows the LSV for Pt/HOPG under the same conditions, and as already pointed out, the peak for the diffusion-limited H_2_ evolution is seen around −0.5 V vs. Ag/AgCl. This peak is shifted with respect to that observed for bulk Pt (−0.4 V, Figure 2), because the surface is not completely covered with Pt, and the electrode material behaves with a rate constant lower than that of Pt. For the Pt/HOPG electrode, k^0^ = 6 ×10^−3^ cm/s, as obtained from DigiElch (Elchsoft, Kleinromstedt, Germany) lower than the bulk Pt k^0^ = 0.36 cm/s [42]. The simulations are shown in Figure 7B (line b) to show the overlap with the experimental data (line a) and were performed based on the previously reported H^+^ diffusion coefficient [42] of D_H_^+^ = 7.1 × 10^−5^ cm^2^/s for H_2_, D_H_2__ = 3.37 ×10^−5^ cm^2^/s [43]. Based on this, a new electrode was prepared with the electrodeposition potential held at −0.5 V vs. Ag/AgCl, which is at the diffusion-limited rate of H_2_ evolution. The AFM image of a sample prepared at −0.5 V vs. Ag/AgCl is depicted in Figure 8. The sample prepared under these conditions presents two populations of Pt structures. In addition to platelets 40 nm in diameter and 3–4 nm thick, larger Pt aggregates (ca. 120 × 30 nm) appears, which are not observed when the sample is prepared at larger overpotentials (−2.0 V) [19,29].

**Figure 9:**
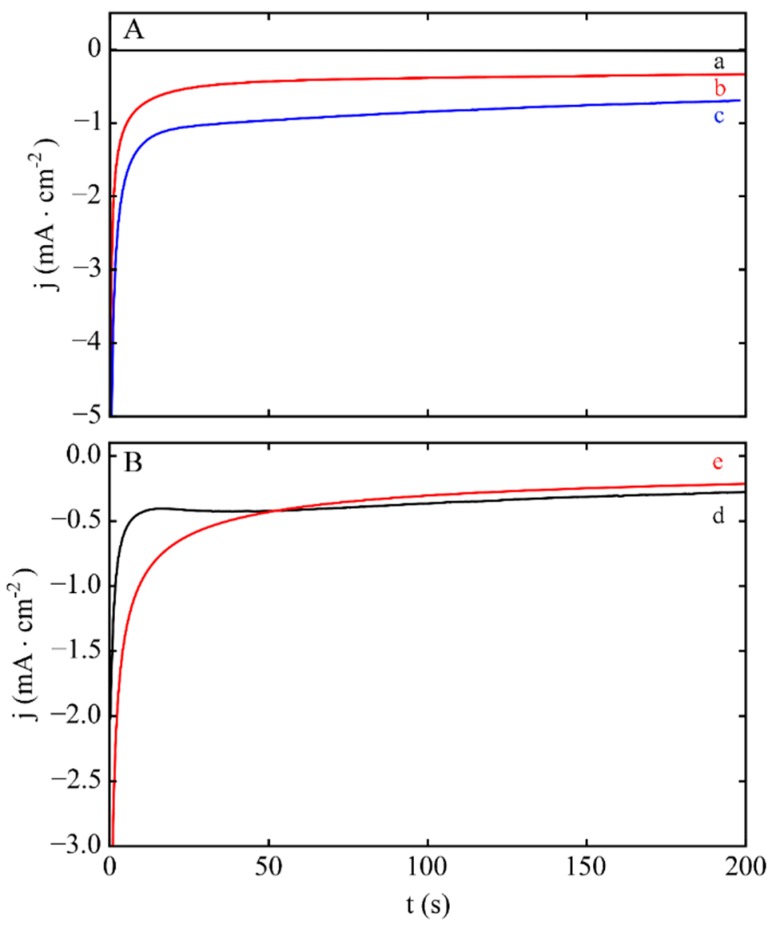
(**A**) Current obtained for the electrodeposition of Pt at E = −0.5 V vs. Ag/AgCl. a: HOPG in blank solution of 0.5 M NaCl, pH 2.3, b: sample deposited with Pt in blank solution, c: Pt deposition in 3 mM H_2_PtCl_6_, 0.5 M NaCl, pH 2.3. (**B**) Pt deposition current (line d) obtained by subtracting lines (a) and (b) from line (c) in (**A**). The (d) line in (**B**) is the experimental curve, while (e) represents the behavior expected from the Cottrell equation.

**Figure 10:**
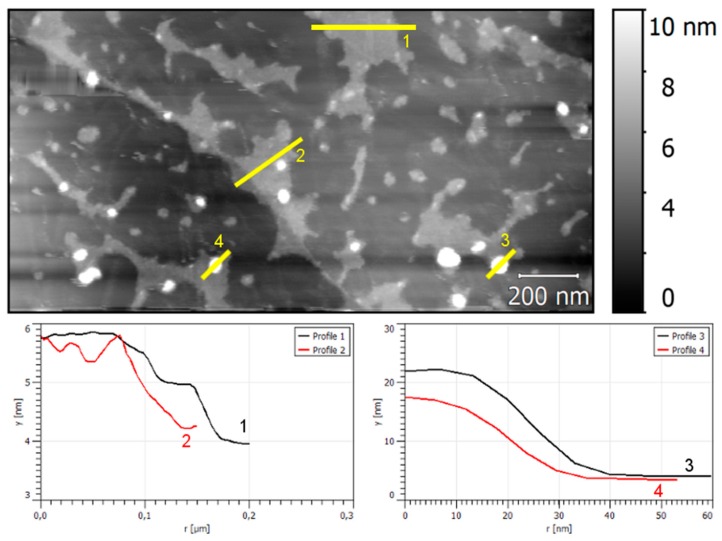
AFM image of a sample electrodeposited at −2 V vs. Ag/AgCl showing the radial profiles measurements of the two populations of deposited structures.

Figure 9 shows the results for the electrodeposition of Pt as a function of time. The current was recorded during deposition, and then, after the Pt deposition was finished, a control experiment was run in the blank solution to study the contribution of H_2_ evolution. The corrected current gives an estimate of the amount of Pt deposited under these conditions. Interestingly, the current at short times (t < 50 s) is much lower than expected from the diffusion behavior (note that it is less negative than the calculation based on the reported Pt diffusion coefficient, D_Pt_^4+^ = 2.2 × 10^−5^ cm^2^/s) [31]. These results are consistent with our observation that in the CVs the diffusion-limited peak is not seen on HOPG. At higher deposition times, the current becomes larger than the expected from diffusion-limited behavior. At this point, enough Pt has been deposited on the surface to drive the formation of H_2_ bubbles that disturb the solution, therefore, increasing the value of the current. Integrating the current of the Pt deposition provides an estimate for the upper limit of Pt deposition, which corresponds to 40 μg/cm^2^. However, under the conditions of high overpotentials (E = −2 V vs. Ag/AgCl), the loading is expected to be much lower based on the size of the Pt features: the Pt NPs in Figure 8 (E = −0.5 V) are much larger than the nanostructures on Figure 10 (E = −2 V) are mostly below 2 nm in high. This sample prepared at high overpotentials shows the nanoplatelets previously described [19,28,29], together with some larger Pt aggregates. The latter are smaller than those seen above obtained at lower overpotentials (Figure 8) (ca. 90 × 20 nm). On the contrary, the nanoplatelets form more extended structures of ca. 200 nm with a 2–6 nm thickness. Therefore, it is likely that H_2_ plays a role in limiting the 3D growth of the Pt deposits and favoring the formation of contiguous Pt islands. Based on the work presented here, we propose that the effect of H_2_ becomes dominant at larger overpotentials, where water electrolysis is predominant over proton reduction.

**Figure 11:**
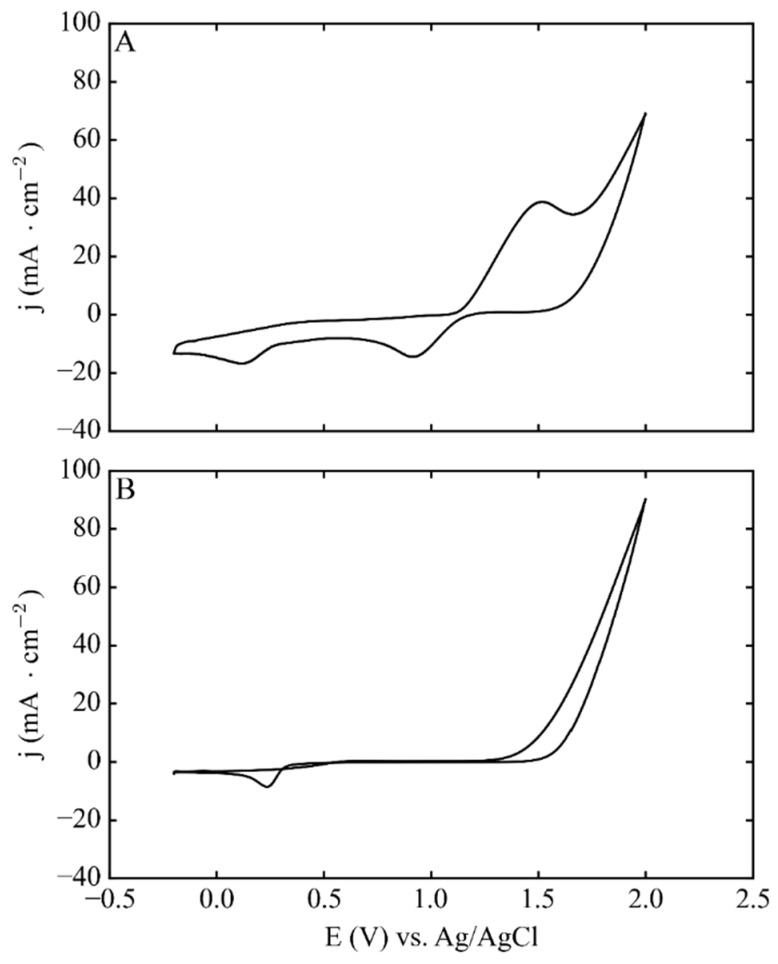
CVs of a 1.6 mm diameter Pt electrode in (**A**) 0.1 M HCl and in (**B**) 0.05 M H_2_SO_4_, ν = 0.5 V/s.

Chloride ions have been proposed to control NP growth [31,32]. A set of CVs of Pt in solutions containing Cl^−^ is shown in Figure 11 recorded at a scan rate ν = 0.5 V/s (A, 0.1 M HCl), and are compared with CVs obtained using a solution of the same pH without Cl^−^ (0.05 M H_2_SO_4_). The scan rate and the potential window were chosen to show the different peaks, given that the processes involved in the PtCl*_x_* reduction overlap with H_2_ evolution. The CVs in HCl show after the formation of PtCl*_x_*, several reduction peaks, around 0.8 V, 0.2 and 0 V vs. Ag/AgCl. The last is likely due to H_2_ processes because it is also present when using the H_2_SO_4_ solution. Therefore, the re-dissolution of Cl^−^ into the solution is proposed to occur in the potential region between 0.8 and 0.2 V vs. Ag/AgCl. These potentials are considerably more positive than those used in the electrodeposition of Pt in this work. However, we should notice that while the stripping of Cl^−^ is seen at more positive potentials, at negative potentials, Cl^−^ could still be present in proximity to the Pt surface, as it is expected to be a major component of the double layer. However, the Cl^−^ will be expected to be weakly bound to the Pt surface under these conditions.

## 4. Conclusions

The electrodeposition conditions for Pt/HOPG were investigated, and it was observed that once the HOPG electrode is immersed in solution, Pt spontaneously deposits on the HOPG surface and forms nanostructures that control the electrodeposition of Pt. This is due to the reduction of PtCl_x_ complexes being kinetically more favorable on the Pt surface than on the HOPG surface. In fact, the HOPG surface activity towards the reduction of Pt-Cl complexes is a strong function of the nature of the surface characteristics, which at the moment cannot be controlled. The defects on the surface drive the deposition of Pt and that the density of the active sites along the surface (defects, edges, etc.) varies depending on the exfoliation of HOPG. The growth of Pt limited to an island morphology is likely due to H_2_ evolution from water reduction, but not from the H^+^ reduction. Thus, a different process that occurs at more negative potentials is likely the cause for the limiting Pt growth. From the CVs it is apparent that, besides the reduction of free H^+^, the reduction of water also is important at potentials more negative than −1 V. Therefore, it is considered that an intermediate in the reduction of water plays a role in protecting the Pt. These features of the electrodeposition are currently being investigated in our laboratories and will be reported in due time.
